# The Role of Endothelial L-PGDS in the Pro-Angiogenic and Anti-Inflammatory Effects of Low-Dose Alcohol Consumption

**DOI:** 10.3390/cells13232007

**Published:** 2024-12-05

**Authors:** Jiyu Li, Chun Li, Utsab Subedi, Pushpa Subedi, Manikandan Panchatcharam, Hong Sun

**Affiliations:** Department of Cellular Biology and Anatomy, LSU Health Shreveport, Shreveport, LA 71103, USA; jiyu.li@lsuhs.edu (J.L.); chun.li@lsuhs.edu (C.L.); utsab.subedi@lsuhs.edu (U.S.); pushpa.subedi@lsuhs.edu (P.S.); manikandan.panchatcharam@lsuhs.edu (M.P.)

**Keywords:** alcohol, L-PGDS, angiogenesis, inflammation, ischemic stroke

## Abstract

Light alcohol consumption (LAC) may reduce the incidence and improve the prognosis of ischemic stroke. Recently, we found that LAC promotes cerebral angiogenesis and inhibits early inflammation following ischemic stroke. In addition, LAC upregulates lipocalin-type prostaglandin D2 synthase (L-PGDS) in the brain. Thus, we determined the role of endothelial L-PGDS in the protective effect of LAC. In in vitro studies, chronic exposure to low-concentration ethanol upregulated L-PGDS and significantly increased the proliferation in cultured C57BL/6J mouse brain microvascular endothelial cells (MBMVECs). AT-56, a selective L-PGDS inhibitor, abolished low-concentration ethanol exposure-induced proliferation. In in vivo studies, 8-week gavage feeding with 0.7 g/kg/day ethanol, defined as LAC, promoted cerebral angiogenesis under physiological conditions and following ischemic stroke in male C57BL/6J mice. In addition, LAC inhibited the post-ischemic expression of adhesion molecules, neutrophil infiltration, and microglial activation. AT-56 and endothelial cell (EC)-specific L-PGDS conditional knockout did not significantly alter cerebral angiogenesis and post-ischemic inflammation in the control mice but eliminated the pro-angiogenic and anti-inflammatory effects of LAC. Furthermore, EC-specific L-PGDS conditional knockout alleviated the neuroprotective effect of LAC against cerebral ischemia/reperfusion injury. These findings suggest that endothelial L-PGDS may be crucial in the pro-angiogenic and anti-inflammatory effects of LAC against ischemic stroke.

## 1. Introduction

Ischemic stroke, which results from a blockage in an artery that supplies the brain, remains one of the leading causes of death and permanent long-term disability worldwide. The blockage results in a reduction of oxygen and nutrients, leading to ischemic death or injury of surrounding brain tissue. Recanalization/reperfusion by intravenous injection of recombinant tissue plasminogen activator (tPA) and endovascular thrombectomy appears critical to limit ischemic brain damage. However, it may paradoxically worsen brain damage through reperfusion injury [1]. Angiogenesis is the generation of new blood vessels from pre-existing vessels and occurs under physiological conditions and several pathological processes, including ischemic stroke [2]. Promoting pre-ischemic angiogenesis may be an effective strategy to reduce brain ischemia/reperfusion (I/R) injury [3], whereas promoting post-ischemic angiogenesis increases not only blood flow but also neurogenesis in the ischemic area and thus improves neurological recovery in both animal models and patients [4,5,6,7]. Interestingly, we recently discovered that light alcohol consumption (LAC) promotes cerebral angiogenesis under physiological conditions and following ischemic stroke [8]. 

On the other hand, post-ischemic inflammation is one of the crucial elements contributing to early cerebral I/R injury [1,9]. It is characterized by several interacting elements, including upregulation of adhesion molecules, recruitment of leukocytes, activation of microglia, and production of matrix metallopeptidases (MMPs), chemokines, and cytokines. The recruitment of neutrophils to the ischemic area, which is facilitated by the upregulation of adhesion molecules on endothelial cells, appears to be a central feature to the reduced regional cerebral blood flow, activation of microglia, release of neurotoxic cytokines, and subsequent neuronal damage [1,10,11,12]. Our previous study found that LAC alleviates post-ischemic inflammatory response and reduces cerebral I/R injury [13].

L-PGDS is the principal PGH_2_ isomerase and is highly expressed in the central nervous and cardiovascular systems [14,15]. A previous study reported that global knockout of L-PGDS significantly increased infarct size following either transient or permanent focal cerebral ischemia [16]. Recently, we found that LAC upregulates L-PGDS in the cerebral cortex. In addition, the L-PGDS antagonist abolished the protective effect of LAC against cerebral I/R injury, and neuron-specific L-PGDS conditional knockout eliminated the inhibitory effect of LAC on neuronal apoptosis following ischemic stroke [17]. Previous studies reported that L-PGDS and its enzymatic products may be involved in inflammation and angiogenesis [18,19]. In the present study; therefore, we specifically determined the role of endothelial L-PGDS in the pro-angiogenic and anti-inflammatory effects of LAC. 

## 2. Methods

### 2.1. Mouse Brain Microvascular Endothelial Cells 

C57/BL6J mouse brain microvascular endothelial cells (C57-6023, CellBiologics, Chicago, IL, USA) were cultured and propagated at 37 °C in a humidified atmosphere of 5% CO_2_ in a medium (M1168, CellBiologics) that contains essential and non-essential amino acids, vitamins, organic and inorganic compounds, trace minerals, fetal bovine serum, growth factors, hormones, antibiotics, and endothelial cell growth supplement. The cells were used between passages 4–8. To examine the chronic effect of alcohol, MBMVECs were exposed to 5 mM and 10 mM ethanol for 2 h or 50 mM ethanol for 4 h once a day for 7 days. To determine the role of L-PGDS, 1 μM AT-56 was added into the medium from the 6th day for two days. Then, a proliferation assay was performed as below. To avoid the possible effect of acute alcohol, the last exposure to ethanol was 24 h before the assay.

### 2.2. Proliferation Assay 

The effect of chronic alcohol exposure on the proliferation of MBMVECs was determined as described previously [8]. Briefly, 7-day 10 mM ethanol-exposed cells were seeded and settled at 37 °C in a humidified atmosphere of 5% CO_2_ for 4 h. Subsequently, the cells were further incubated for 2 h after adding 10 µL of Cell Counting Kit-8 (Dojindo Molecular Technologies, Rockville, MD, USA) reagent to each well. The absorbance at 450 nm was measured with the FLUOstar Omega microplate reader (BMG LABTECH, Ortenberg, Germany) and expressed as percentage changes to the control. 

### 2.3. Animal Models

All of the procedures and protocols were preapproved by the Institutional Animal Care and Use Committee (IACUC) at the Louisiana State University (LSU) Health Shreveport and performed following the ARRIVE (Animal Research: Reporting in Vivo Experiments) guidelines and the National Institutes of Health Guide for the Care and Use Laboratory Animals. Sixty-two male wild-type (WT) C57BL/6J mice (3 months) (25–30 g) were randomly divided into three groups and gavage fed with 0.7 g/kg (defined as LAC, *n* = 29), 2.8 g/kg (defined as HAC, *n* = 4) ethanol, or volume-matched water (defined as control, *n* = 29) once a day for 8 weeks. From the 5th week, 16 mice from the control and LAC groups were orally given AT-56 (30 mg/kg/day) for 4 weeks. In addition to WT mice, 73 Tie2^CreERT2/+^/L-PGDS^fl/fl^ and 47 L-PGDS^fl/fl^, and 22 Tie2^CreERT2/+^/L-PGDS^+/+^ mice on a C57BL/6J background were randomly divided into two groups and gavage fed with 0.7 g/kg ethanol or volume-matched water once a day for 8 weeks. From the 6th week, all Tie2^CreERT2/+^/L-PGDS^fl/fl^, L-PGDS^fl/fl^, and Tie2^CreERT2/+^/L-PGDS^+/+^ mice were treated with tamoxifen (0.5 mg/kg/day, IP) for 3 weeks. At the end of 8 weeks of feeding, body weight, mean arterial blood pressure (MABP), heart rate, and fasting blood glucose were measured as described previously [13]. From each group, 4–5 mice were euthanized to measure vessel density of the brain and expression of L-PGDS under physiological conditions. 

### 2.4. Transient Focal Cerebral Ischemia 

From each group, five mice were subjected to transient focal cerebral ischemia for measuring cerebral I/R injury, neutrophil infiltration, and microglial activation, 4–7 mice were subjected to transient focal cerebral ischemia for measuring ICAM-1 and E-selectin expression and 4–5 mice were subjected to transient focal cerebral ischemia for measuring post-ischemic cerebral angiogenesis. Transient focal cerebral ischemia was induced by unilateral middle cerebral artery occlusion (MCAO) for 90 or 60 min, as described previously [13]. To avoid the potential influence of acute alcohol, ethanol was not given on the day before the experiment. The mouse was anesthetized with isoflurane (induction at 5% and maintenance at 1.5%) in a gas mixture containing 30% O_2_/70% N_2_. The body temperature was maintained with a temperature-controlled heating pad (Harvard Apparatus, March, Germany) during the procedure, and regional cerebral blood flow (rCBF) of the right MCA territory was monitored by a Laser-Doppler flow probe (PERIMED, PF 5010 LDPM Unit, Jarfalla, Sweden). To occlude the right MCA, the right common carotid artery (CCA) and external carotid artery (ECA) were exposed and ligated. Subsequently, a silicon rubber-coated monofilament (Doccol Corporation, Sharon, MA, USA) was inserted from the basal part of the right ECA and advanced cranially into the right internal carotid artery (ICA) to the point where the ICA bifurcates into the MCA and the anterior cerebral artery (ACA). A rapid drop in rCBF indicated the onset of MCAO. After a 90 or 60 min occlusion, the monofilament was removed, and the CCA was reopened to achieve reperfusion. Mice with 90 min MCAO were euthanized at 24 h of reperfusion for measuring cerebral I/R injury, ICAM-1 and E-selectin expression, neutrophil infiltration, and microglial activation. Mice with 60 min MCAO were euthanized at 72 h and 7 days of reperfusion for evaluating post-ischemic angiogenesis. All mice were euthanized with 5% isoflurane in a mixture of 30% O_2_ and 70% N_2_ via a chamber of the vaporizer, followed by open-chest exsanguination or transcardiac perfusion with phosphate-buffered saline (PBS) [13].

### 2.5. Western Blot Analysis 

To measure the expression of L-PGDS, ICAM-1, and E-selectin, the cerebral cortex, peri-infarct cerebral cortex, and 7-day ethanol-treated MBMVECs were homogenized in an ice-cold lysis buffer (pH 7.4), which contains 1% Triton, 0.1% mercaptoethanol, 10 mM EDTA, 0.1% Tween-20, 150 mM NaCl, 50 mM Tris HCl, 5 μg/mL leupeptin, 0.1 mM phenylmethylsulfonyl fluoride, and 5 μg/mL aprotinin. Homogenates were centrifuged (12,000 RPM) at 4 °C for 20 min, and the supernatants were collected. The protein concentration of the supernatants was determined using the Bradford method (Bio-Rad, CA, USA). After the protein concentration was adjusted, a 10% gel was used to conduct SDS polyacrylamide gel electrophoresis (SDS-PAGE). Following SDS-PAGE, the proteins were transferred from the gel onto the polyvinylidene difluoride membrane. Immunoblotting was performed using rabbit anti-L-PGDS (Novus, Littleton, CO, USA), mouse anti-ICAM-1 (Santa Cruz Biotechnology), and rabbit anti-E-selectin (Abcam) as the primary and peroxidase-conjugated goat anti-mouse and goat anti-rabbit IgG as the second antibody. As described previously [13], The target proteins were subsequently detected with the enhanced chemiluminescence (ECL) kit (Pierce Chemical, Dallas, TX, USA). The band densities were analyzed using the ChemiDoc^TM^ MP Imaging System (Bio-Rad, Hercules, CA, USA). For quantification, protein expression of L-PGDS, ICAM-1, and E-selectin were normalized to GAPDH or β-actin and expressed as percentage changes to the control. 

### 2.6. Immunohistochemistry Staining 

To examine the effects of LAC on cerebral I/R injury, neutrophil infiltration, microglial activation, and cerebral angiogenesis, mice were anesthetized and subjected to transcardiac perfusion with 25 mL PBS and 20 mL 4% paraformaldehyde. The brains were removed, fixed in 4% paraformaldehyde overnight, dehydrated in a graded series of sucrose solutions for 72 h, then embedded in O.C.T. compound (Fisher Scientific, Hampton, NH, USA), and quickly frozen in liquid nitrogen for 5 min. The frozen brains were sliced into 14 μm coronal sections and placed on frost-free slides. Three sections (1.21 mm rostral and 0.23 mm and 1.31 mm caudal to bregma) from each mouse were washed with PBS, blocked with a mixture of 0.1% Trypsin and 10% bovine serum albumin (BSA) for 1 h, and then incubated with 1:100 goat anti-CD31 antibody (R&D Systems, Minneapolis, MN, USA), 1:100 rabbit anti-myeloperoxidase (MPO) (Abcam, Cambridge, UK) or 1:100 rabbit anti-ionized calcium-binding adaptor molecule 1 (Iba1) (Wako Chemicals Inc., Richmond, VA, USA) at 4 °C overnight. Following washes with PBS, as described previously [13], the sections were incubated with 1:200 biotinylated rabbit anti-goat lgG antibody (Vector Labs, Newark, CA, USA) for 1 h, then incubated with 1:200 streptavidin Alexa Fluor™ 488 conjugate (Thermo Fisher, Waltham, MA, USA) or 1:200 AlexaFluor 555 donkey anti-rabbit (Santa Cruz Technology, Dallas, TX, USA) for 30–60 min at room temperature. Following washes, sections were covered with a DAPI mounting medium (Vector Labs) and observed using a fluorescence microscope (Nikon Eclipse Ts2, Nikon Instruments Inc., Melville, NY, USA). For quantitative analysis, four images were taken from each region of interest. CD31 intensity and the number of branches were quantified using NIS-Elements software (BR 4.51.00, Nikon Instruments Inc.) and expressed as percentage changes to the control. Cells positive for MPO represented infiltrating neutrophils. For quantitative analysis, positive cells were counted in three separate areas per section surrounding the infarct area. To quantify microglia, we meticulously observed and counted cells that tested positive for Iba1, a specific marker for these cells. In their resting state, microglia have long, branching processes extending from their cell bodies, giving them a highly ramified appearance [20]. This structure allows them to survey their environment efficiently. However, when activated, microglia undergo a significant change in shape. They shift from their branched form to a more amoeboid shape, ultimately losing their processes. This change in shape is accompanied by an increase in Iba1 expression, indicating their activated status [21]. For our study, we classified microglia as activated if they showed elevated Iba1 expression and had three or fewer processes. We carefully developed this classification criterion, which enabled us to distinguish between resting and activated microglia with precision.

### 2.7. Nissl Staining

Brain sections ranging from 2.9 mm anterior to 4.9 mm posterior to the bregma were selected from four specific coronal levels to measure cerebral infarct size as previously described [22,23]. These sections were subjected to Nissl staining using a 0.01% cresyl violet acetate solution (IHC World, Ellicott City, MD, USA) for 5 min at room temperature. Post-staining, the sections were rinsed in distilled water, dehydrated through a graded ethanol series, cleared in xylene, and mounted with a xylene-based medium from VWR. The stained sections were photographed at 1.0× magnification with an Olympus microscope. ImageJ software (1.54f) was used to analyze the images, identifying infarct lesions as areas lacking Nissl stain. The infarct size was calculated by integrating the location of these lesions across the different levels and expressing the total as a percentage of the hemisphere’s volume. This method provides an accurate and reliable measurement of infarct size, which is crucial for understanding the pathological effects of cerebral ischemia. 

### 2.8. Statistical Analysis

All the quantitative data are presented as means ± standard deviation (SD). Prism 9 (GraphPad, Boston, MA, USA) was used for statistical analyses. The difference in L-PGDS expression was analyzed with one-way ANOVA followed by Dunnett’s test. The difference in proliferation of MBMVECs, CD31 intensity and number of branches, infarct size, ICAM-1 and E-selectin expression, neutrophil infiltration, and microglial activation was analyzed with two-way ANOVA followed by Tukey’s test. The differences are considered statistically significant as the *p*-value is less than 0.05.

## 3. Results

### 3.1. Effect of Chronic Alcohol Exposure on Expression of L-PGDS and Angiogenic Capability in MBMVECs

**Figure 1:**
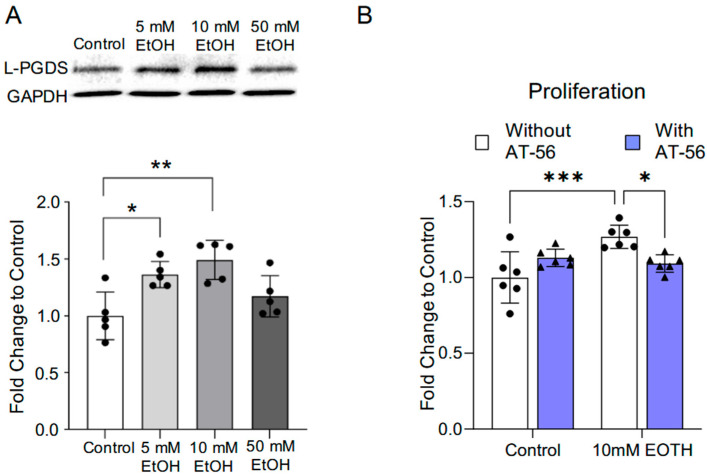
The influence of chronic alcohol exposure on L-PGDS protein expression and proliferation in MBMVECs. (**A**) Representative Western blots of L-PGDS (upper). Values are means ± SD (lower, *n* = 5). * *p* < 0.05, ** *p* < 0.005. Analyzed using one-way ANOVA with Dunnett’s post hoc. (**B**) Values are means ± SD (*n* = 6) for proliferation in the Proliferation Assay. * *p* < 0.05, *** *p* < 0.0005. Analyzed using two-way ANOVA followed by Tukey’s test.

Two-hour exposure to 5 mM and 10 mM ethanol daily for 7 days upregulated L-PGDS in MBMVECs. In contrast, four-hour exposure to 50 mM once a day for 7 days did not significantly change L-PGDS protein expression (Figure 1A). On the other hand, 2 h exposure to 10 mM ethanol once a day for 7 days significantly increased the proliferation of MBMVECs in the Proliferation Assay (Figure 1B). Treatment with 1 μM AT-56 from the 6th day for two days did not significantly change proliferation in control MBMVECs but eliminated 10 mM ethanol exposure-induced proliferation (Figure 1B). 

### 3.2. Physiological Parameters 

As shown in Table 1, there was no significant difference in physiological parameters, including body weight, mean arterial blood pressure (MABP), heart rate, and fasting blood glucose level among water-fed and 0.7 g/kg/day ethanol-fed L-PGDS^fl/fl^ and Tie2^CreERT2/+^/L-PGDS^fl/fl^ mice.

### 3.3. LAC-Induced Cerebral Angiogenesis Under Physiological Conditions

**Figure 2:**
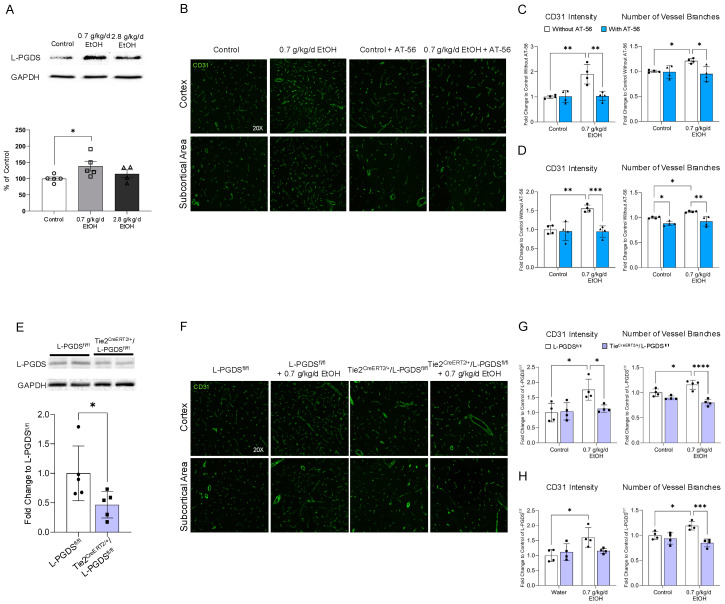
The influence of chronic alcohol exposure on L-PGDS protein expression in the cerebral cortex and cerebral angiogenesis under physiological conditions. (**A**) Representative Western blots of L-PGDS (upper). Values are means ± SD (lower, *n* = 4–5) for L-PGDS expression in the cerebral cortex. * *p* < 0.05. Analyzed using one-way ANOVA with Dunnett’s post hoc. (**B**) Representative images of CD31 staining. (**C**) Values are means ± SD (*n* = 4) for CD31 intensity and the number of vessel branches in the cerebral cortex. (**D**) Values are means ± SD (*n* = 4) for CD31 intensity and the number of vessel branches in the subcortical area. * *p* < 0.05, ** *p* < 0.005, *** *p* < 0.0005. Analyzed using two-way ANOVA followed by Tukey’s test. (**E**) Representative Western blots (upper). Values are means ± SD (lower, *n* = 5) for L-PGDS expression in the lung. * *p* < 0.05. Analyzed using unpaired *t*-test. (**F**) Representative images of CD31 staining. (**G**) Values are means ± SD (*n* = 4) for CD31 intensity and the number of vessel branches in the cerebral cortex. (**H**) Values are means ± SD (*n* = 4) for CD31 intensity and the number of vessel branches in the subcortical area. * *p* < 0.05, *** *p* < 0.0005, **** *p* < 0.0001. Analyzed using two-way ANOVA followed by Tukey’s test.

As shown in Figure 2A, 8-week daily feeding with 0.7 g/kg/day ethanol upregulated L-PGDS, whereas 2.8 g/kg/day ethanol did not significantly alter L-PGDS expression in the cerebral cortex. Similarly to the previous report [8], 8-week feeding with 0.7 g/kg/day ethanol significantly increased CD31 intensity and the number of vessel branches in the cerebral cortex and subcortical area of WT mice under physiological conditions. Four-week treatment with 30 mg/kg/day AT-56 significantly reduced the number of vessel branches in the subcortical region of control WT mice. It abolished the 0.7 g/kg/day ethanol-induced increase in CD31 intensity and vessel branches in the cerebral cortex and subcortical area (Figure 2B–D). As shown in Figure 2E, protein expression of L-PGDS in the lung was significantly reduced in 3-week tamoxifen-treated Tie2^CreERT2/+^/L-PGDS^fl/fl^ mice compared to tamoxifen-treated L-PGDS^fl/fl^ mice, indicating the success of L-PGDS conditional knockout in the endothelial cells. Similarly to those observed in the WT mice, 8-week feeding with 0.7 g/kg/day ethanol significantly increased CD31 intensity and vessel branches of the cerebral cortex and subcortical area in L-PGDS^fl/fl^ mice under physiological conditions. However, such an increase could not be observed in 0.7 g/kg/day ethanol-fed Tie2^CreERT2/+^/L-PGDS^fl/fl^ mice (Figure 2F–H).

### 3.4. Protein Expression of Adhesion Molecules

**Figure 3:**
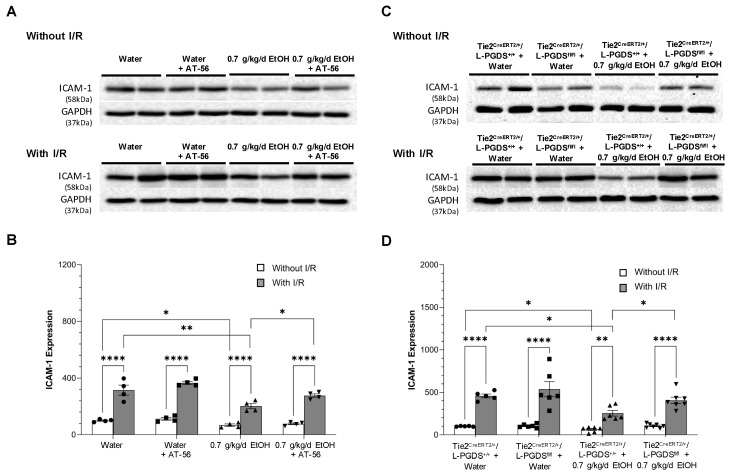
The effect of AT-56 and EC-specific L-PGDS knockout on ICAM-1 expression in the cerebral cortex under physiological conditions and following ischemic stroke. (**A**,**C**) Representative Western blots. (**B**,**D**) Values are means ± SD for 4–7 mice in each group. * *p* < 0.05, ** *p* < 0.005, **** *p* < 0.0001. Analyzed using two-way ANOVA followed by Tukey’s test.

**Figure 4:**
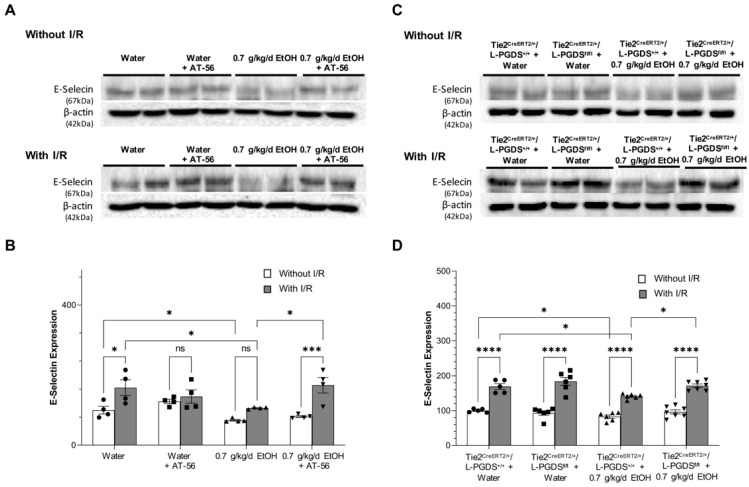
The effect of AT-56 and EC-specific L-PGDS knockout on E-selectin expression in the cerebral cortex under physiological conditions and following ischemic stroke. (**A**,**C**) Representative Western blots. (**B**,**D**) Values are means ± SD for 4–7 mice in each group. * *p* < 0.05, *** *p* < 0.0005, **** *p* < 0.0001. Analyzed using two-way ANOVA followed by Tukey’s test.

Eight-week intake of 0.7 g/kg/day ethanol slightly but significantly downregulated baseline expression of ICAM-1 (Figure 3A,B) and E-selectin (Figure 4A,B) in WT mice. Overall, 90 min MCAO/24 h reperfusion significantly upregulated ICAM-1 in control and ethanol-fed WT mice. However, the magnitude of upregulation was considerably less in ethanol-fed WT mice (Figure 3B). In addition, 90 min MCAO/24 h reperfusion significantly upregulated E-selectin in control but not ethanol-fed WT mice (Figure 4A,B). AT-56 administration prevented post-ischemic upregulation of E-selectin in control WT mice. Moreover, it abolished the inhibitory effect of 0.7 g/kg/day ethanol on ICAM-1 and E-selectin expression under physiological conditions and following ischemic stroke (Figure 3A,B and Figure 4A,B). On the other hand, the inhibitory effect of 0.7 g/kg/day ethanol on the baseline and post-ischemic expression of ICAM-1 and E-selectin was also observed in Tie2^CreERT2/+^/L-PGDS^+/+^ mice (Figure 3C,D and Figure 4C,D). However, the inhibitory effect of ethanol on both ICAM-1 and E-selectin under physiological conditions and following ischemic stroke was diminished in Tie2^CreERT2/+^/L-PGDS^fl/fl^ mice (Figure 3C,D and Figure 4C,D).

### 3.5. Infarct Size, Neutrophil Infiltration, and Microglial Activation

**Figure 5:**
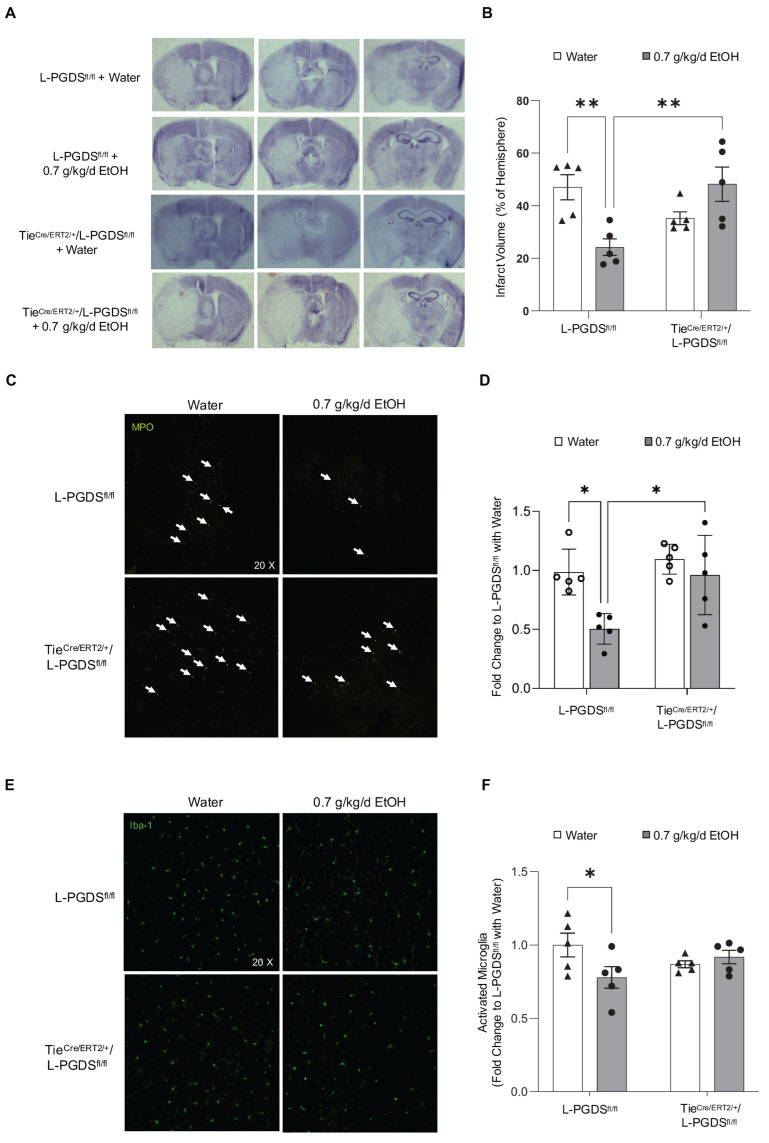
The effect of EC-specific L-PGDS knockout on infarct size and neutrophil infiltration and microglial activation in the peri-infarct cortex at 24 h of reperfusion following a 90 min MCAO. (**A**) Representative brain sections stained with cresyl violet. (**B**) Values are means ± SD for 5 mice in each group. (**C**) Representative immunohistochemistry staining of MPO. (**D**) Values are means ± SD for 5 mice in each group. (**E**) Representative immunohistochemistry staining of Iba1. (**F**) Values are means ± SD for 5 mice in each group. * *p* < 0.05, ** *p* < 0.005. Analyzed using two-way ANOVA followed by Tukey’s test.

We previously reported that the 4-week administration of AT-56 attenuated the protective effect of LAC against cerebral I/R injury [17]. In the present study, we further measured the infarct size in water- and 0.7 g/kg/day ethanol-fed L-PGDS^fl/fl^ and Tie2^CreERT2/+^/L-PGDS^fl/fl^ mice. As shown in Figure 5A,B, 8-week feeding with 0.7 g/kg/day ethanol significantly reduced infarct size at 24 h of reperfusion following a 90 min MCAO in L-PGDS^fl/fl^ mice. EC-specific L-PGDS conditional knockdown did not alter 90 min MCAO/24 h reperfusion-induced cerebral damage in water-fed mice but significantly diminished the neuroprotective effect of LAC against cerebral I/R injury. We further examined post-ischemic neutrophil infiltration and microglial activation in L-PGDS^fl/fl^ and Tie2^CreERT2/+^/L-PGDS^fl/fl^ mice. As shown in Figure 5C–F, 90 min MCAO/24 h reperfusion led to neutrophil infiltration and microglial activation in the peri-infarct cortex of both L-PGDS^fl/fl^ and Tie2^CreERT2/+^/L-PGDS^fl/fl^ mice. Compared to water-fed L-PGDS^fl/fl^ mice; however, the magnitude of neutrophil infiltration (Figure 5C,D) and microglial activation (Figure 5E,F) was significantly less in 0.7 g/kg/day ethanol-fed L-PGDS^fl/fl^ mice. In contrast, the difference was not observed between water- and ethanol-fed Tie2^CreERT2/+^/L-PGDS^fl/fl^ mice (Figure 5C–F). However, neutrophil infiltration was significantly increased in ethanol-fed Tie2^CreERT2/+^/L-PGDS^fl/fl^ mice compared to ethanol-fed L-PGDS^fl/fl^ mice (Figure 5C,D).

### 3.6. LAC-Induced Cerebral Angiogenesis Following Ischemia/Reperfusion 

**Figure 6:**
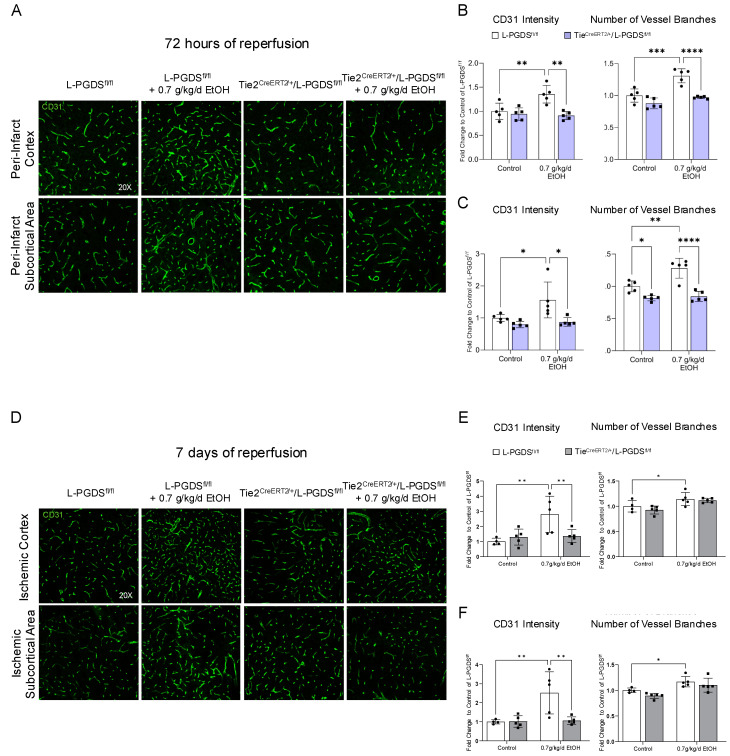
The effect of EC-specific L-PGDS conditional knockout on LAC-induced cerebral angiogenesis at 72 h and 7 days of reperfusion following a 60 min ischemic stroke. (**A**) Representative images of CD31 staining at 72 h of reperfusion. (**B**) Values are means ± SD (*n* = 5) for CD31 intensity and the number of vessel branches in the peri-infarct cortex. (**C**) Values are means ± SD (*n* = 5) for CD31 intensity and the number of vessel branches in the peri-infarct subcortical area. (**D**) Representative images of CD31 staining at 7 days of reperfusion. (**E**) Values are means ± SD (*n* = 4–5) for CD31 intensity and the number of vessel branches in the ischemic cortex. (**F**) Values are means ± SD (*n* = 4–5) for CD31 intensity and the number of vessel branches in the ischemic subcortical area. * *p* < 0.05, ** *p* < 0.005, *** *p* < 0.0005, **** *p* < 0.0001. Analyzed using two-way ANOVA followed by Tukey’s test.

Overall, 60 min MCAO increased CD31 intensity and the number of vessel branches in the peri-infarct cortex and subcortical area at 72 h and 7 days of reperfusion (Figure 6). However, the magnitude of increase was significantly greater in 0.7 g/kg/day ethanol-fed L-PGDS^fl/fl^ mice compared to the control L-PGDS^fl/fl^ mice (Figure 6B,C,E,F). In addition, the ethanol-induced increase in CD31 intensity and the number of vessel branches was not observed in Tie2^CreERT2/+^/L-PGDS^fl/fl^ mice at 72 h of reperfusion (Figure 6B,C). Interestingly, although the ethanol-induced increase in CD31 intensity was not found, the ethanol-induced increase in the number of vessel branches remained in Tie2^CreERT2/+^/L-PGDS^fl/fl^ mice at 7 days of reperfusion (Figure 6E,F). 

## 4. Discussion

The present study investigated the possible role of endothelial L-PGDS in the pro-angiogenic and anti-inflammatory effects of LAC. There are several new findings. First, chronic exposure to low-concentration alcohol upregulated L-PGDS and L-PGDS antagonists inhibited alcohol-induced proliferation in MBMVECs. Second, L-PGDS antagonist and EC-specific L-PGDS conditional knockout abolished LAC-induced cerebral angiogenesis under physiological conditions. Third, L-PGDS antagonists and EC-specific L-PGDS conditional knockout attenuated the inhibitory effect of LAC on the expression of ICAM-1 and E-selectin. Fourth, EC-specific L-PGDS conditional knockout suppressed the protective effect of LAC against cerebral I/R injury. Fifth, EC-specific L-PGDS conditional knockout eliminated the inhibitory effect of LAC on post-ischemic neutrophil infiltration. Sixth, EC-specific L-PGDS conditional knockout significantly inhibited LAC-induced cerebral angiogenesis following ischemic stroke. We speculate that LAC may promote cerebral angiogenesis, inhibit post-ischemic inflammation, and protect the brain against its I/R injury via upregulation of endothelial L-PGDS. 

We previously found that feeding mice with 0.7 g/kg ethanol via a gavage produced a peak blood alcohol concentration of 9 mM, which is usually observed in a man with average body weight (70 kg) following intake of one and a half American standard drinks (14 g of ethanol/each). On the other hand, the peak blood alcohol concentration in mice after gavage feeding with 2.8 g/kg ethanol was 37 mM, which can be found following consumption of a little more than seven American standard drinks [13,24,25]. In the clinic, physicians define “light” drinking as 1.2 drinks/day, “moderate” drinking as 2.2 drinks/day, and “heavy” drinking as 3.5 drinks/day [26]. Thus, 0.7 g/kg/day and 2.8 g/kg/day of ethanol were considered as LAC and HAC in the present study, respectively. Furthermore, 5 mM and 10 mM ethanol were selected as low concentrations and 50 mM ethanol was considered a high concentration in our cell culture studies. Our previous study found that blood alcohol concentration reached zero in LAC mice and remained at about 30 mM in HAC mice at 2 h of gavage feeding [13]. In cell culture experiments, therefore, we chose 2 h and 4 h incubation durations for low- and high-concentration ethanol, respectively. 

L-PGDS is the principal PGH_2_ isomerase, expressed mainly in the central nervous system (CNS), cardiovascular system, and male genital organs [14,15]. L-PGDS catalyzes the conversion of PGH_2_ to PGD_2_, the most abundant prostaglandin produced in the brain [27]. L-PGDS, also known as β-trace in the CNS, acts as an amyloid β chaperone [28]. Previous studies reported that L-PGDS localized in oligodendrocytes, choroid plexus epithelial cells, and leptomeningeal cells [29]. However, neurons may also be a primary cellular source of L-PGDS [17]. L-PGDS appears neuroprotective under various pathological conditions [16,30,31]. Global L-PGDS knockout mice had larger infarcts following ischemic stroke than WT mice [16]. In addition, L-PGDS is involved in the neuroprotective effect of dexamethasone against hypoxic–ischemic brain damage [32]. Unfortunately, the cellular mechanisms underlying the L-PGDS-mediated neuroprotective effect remain unclear. Recently, we found that LAC upregulates L-PGDS in the cerebral cortex. Moreover, AT-56, a selective L-PGDS antagonist, and neuron-specific L-PGDS conditional knockout did not induce neuronal apoptosis under physiological conditions but abolished the inhibitory effect of LAC on post-ischemic neuronal apoptosis [17]. 

In the present study, AT-56 and EC-specific L-PGDS conditional knockout significantly suppressed the inhibitory effect of LAC on ICAM-1 and E-selectin expression. Furthermore, EC-specific L-PGDS conditional knockout abolished the beneficial effect of LAC against post-ischemic neutrophil infiltration and cerebral I/R injury. Generally, PGH_2_ can be converted to TXA_2_, PGI_2_, PDF_2α_, PGE_2_, and PGD_2_. Among these lipid autacoids, PGE_2_ is a principal mediator of inflammation. It induces ICAM-1 expression in the brain endothelial cells and is implicated in leukocyte adhesion and infiltration into the inflamed tissue [33]. In addition, PGE_2_ has been shown to contribute to cell injury in ischemic stroke and neurodegenerative diseases [34,35]. In contrast, PGD_2_ appears to function as an anti-inflammatory lipid mediator. Interestingly, L-PGDS-mediated PGD_2_ synthesis inhibits E-selectin generation in human vascular endothelial cells [18]. Thus, it is conceivable that there may be an imbalance in the production of PGE_2_ and PGD_2_ during LAC. The upregulated L-PGDS leads to an increased PGD_2_ and a reduced PGE_2_ production and subsequently downregulates ICAM-1 and E-selectin in the endothelial cells. Although the function of microglial activation is not fully elucidated, it may play a critical role in neuroinflammation and pathological progression of ischemic brain tissue during the acute phase of ischemic stroke. Several events initially lead to microglial activation, including reduced cerebral blood flow, brain injury, and infection [36]. In addition, cytokines may promote microglial activation [37]. In the present study, EC-specific L-PGDS conditional knockout failed to increase microglial activation significantly. PGD_2_ inhibits cytokine production to suppress allergic reactions [38]. In addition, PGD_2_ inhibits the expression of pro-inflammatory genes in atherosclerosis [39,40]. Furthermore, the non-enzymatic metabolites of PGD_2_, such as 15d-PGJ_2_, 15d-PGD_2_, and Δ^12^-PGJ_2_, are ligands of PPARγ [41]. Our recent studies revealed that LAC increased nuclear PPARγ expression and DNA-binding activity in the cerebral cortex [22,42]. The PPARγ signaling pathway activation attenuates ICAM-1 expression, pro-inflammatory cytokine release, and reactive oxygen species (ROS) production [43,44]. Importantly, treatment with PPARγ ligands reduces microglial activation [44]. Thus, it seems likely that reduced microglial activation during LAC is not related to endothelial L-PGDS upregulation. However, the inhibitory effect of LAC on the expression of adhesion molecules and neutrophil infiltration may be associated with endothelial L-PGDS-mediated production of PGD_2_ and activation of the PPARγ signaling pathway.

AT-56 and EC-specific L-PGDS conditional knockout abolished LAC-induced cerebral angiogenesis under physiological conditions in the present study. In addition, EC-specific L-PGDS conditional knockout significantly suppressed the stimulative effect of LAC on post-ischemic cerebral angiogenesis. As far as we are aware, only a few studies investigated the role of L-PGDS in angiogenesis. An early study reported that L-PGDS may be a genetic locus controlling VEGF-A-mediated angiogenesis [45]. Interestingly, LAC upregulates VEGF-A and VEGFR2 in the cerebral cortex [8]. Recently, a study found that systemic L-PGDS deficiency impaired vessel elongation and sprout redocumentation in neonatal retinal angiogenesis, suggesting the involvement of L-PGDS in physiological angiogenesis [19]. In contrast, endothelial L-PGDS deficiency accelerated tumor vascular hyperpermeability and angiogenesis [46]. On the other hand, systemic L-PGDS deficiency accelerated age-related osteoarthritis but did not alter subchondral bone angiogenesis [47]. The reasons for the discrepancies between these studies are not entirely clear but may be related to the region of the body examined, the age of the animal models, benign and malignant differences in the tissue, and research approaches. Although PGD_2_ may function as a suppressor of angiogenesis [48], the PPARγ signaling pathway, activated by the non-enzymatic metabolites of PGD_2_, may promote angiogenesis. Loss of PPARγ in ECs leads to impaired angiogenesis [49]. In cardiac myofibroblasts, PPARγ activation was reported to upregulate VEGF-A and VEGFR2 and boost angiogenic potential [50]. In adipocytes, the promoter activity of VEGF-A was elevated by PPARγ transfection [51]. Moreover, pretreatment with PPARγ agonist increased angiogenesis, reduced infarct size, and improved functional recovery in rats following transient focal cerebral ischemia [52]. Endogenous lipid mediators of PPARγ enhanced VEGF production and subsequent intramedial angiogenesis in early atheromatous aortas [53]. Thus, we speculate that LAC-induced cerebral angiogenesis is related to upregulated endothelial L-PGDS and L-PGDS-mediated activation of the PPARγ signaling pathway. Following ischemic stroke, angiogenesis in the peri-infarct area reaches statistically significant at 72 h and continues for more than 3 weeks [54]. In light of the previous study, we selected 72 h and 7 days of reperfusion to measure post-ischemic angiogenesis in the present study. Interestingly, although EC-specific L-PGDS conditional knockout significantly reduced CD31 intensity at 72 h and 7 days of reperfusion, it only significantly reduced the number of vessel branches at 72 h in LAC mice. The explanation for this finding remains unclear. Following the 60 min MCAO, both ethanol and tamoxifen were not given. We cannot rule out the possibility that progenitor cells induce a rapid endothelial turnover and repair. In addition to the VEGF signaling pathway, many other cellular and molecular mechanisms are involved in post-ischemic cerebral angiogenesis [55]. Thus, the impact of LAC on other pathways underlying post-ischemic cerebral angiogenesis needs to be further determined.

There are several limitations in the present study. First, alcohol is converted to acetaldehyde by alcohol dehydrogenase (ADH) and subsequently broken down into acetate by aldehyde dehydrogenase (ALDH). The impacts of alcohol metabolites on the expression of L-PGDS, adhesion molecules, and growth factors cannot be ruled out in the present study. Thus, the pro-angiogenic and anti-inflammatory effects of LAC may be altered in ALDH2 deficiency, a genetic condition present in about 8% of the global population. Second, the precise mechanism underlying LAC-induced L-PGDS upregulation and molecular signaling pathways involved in L-PGDS-mediated angiogenesis and inhibitory effect on adhesion molecules remains unknown. Third, AT-56 only significantly reduced the number of vessel branches in the subcortical area, but it failed to alter cortical CD31 intensity, the number of vessel branches, and the expression of adhesion molecules in the control mice. It remains unclear whether it was because of the dose or administration duration of AT-56 or the limited influence of L-PGDS under physiological conditions. 

In summary, we examined the effect of L-PGDS antagonist and EC-specific L-PGDS conditional knockout on cerebral angiogenesis, adhesion molecular expression, neutrophil infiltration, microglial activation, and cerebral I/R injury in LAC mice. LAC upregulated L-PGDS in the cerebral cortex and cultured endothelial cells. In addition, L-PGDS antagonists and EC-specific L-PGDS conditional knockout significantly attenuated the inhibitory effect of LAC on ICAM-1 and E-selectin expression, post-ischemic neutrophil infiltration, and cerebral I/R injury. Furthermore, L-PGDS antagonists and EC-specific L-PGDS conditional knockout significantly alleviated the pro-angiogenic effect of LAC. Therefore, we speculate that upregulated endothelial L-PGDS may contribute to the anti-inflammatory, pro-angiogenic, and neuroprotective effects of LAC. In the future, a better understanding of how alcohol regulates L-PGDS expression will not only result in new approaches for preventing and treating ischemic stroke and neurodegenerative diseases in non-drinkers but also improve the clinical treatment of ischemic stroke in alcohol users. 

## Figures and Tables

**Table 1:** Physiological parameters.

	L-PGDS^fl/fl^ + Water	L-PGDS^fl/fl^ + 0.7 g/kg/day EtOH	Tie2^CreERT2/+^/L-PGDS^fl/fl^ +Water	Tie2^CreERT2/+^/L-PGDS^fl/fl^ + 0.7 g/kg/day EtOH
Body Weight (g)	28.3 ± 3.5	29.9 ± 6.7	28.3 ± 4.5	30.3 ± 5.3
MABP (mmHg)	90.4 ± 16.3	85.6 ± 19.3	91.7 ± 11.9	75.4 ± 14.1
Heart Rate (bpm)	610.7 ± 123.0	631.4 ± 24.2	647.7 ± 48.0	581.2 ± 79.9
Fasting Blood Glucose (mg/dl)	131.0 ± 36.3	123.8 ± 22.9	171.6 ± 44.3	138.7 ± 27.7

## Data Availability

Original raw data is available upon request.
