# Correction: Li et al. The Role of Endothelial L-PGDS in the Pro-Angiogenic and Anti-Inflammatory Effects of Low-Dose Alcohol Consumption. *Cells* 2024, *13*, 2007

**DOI:** 10.3390/cells15010064

**Published:** 2025-12-30

**Authors:** Jiyu Li, Chun Li, Utsab Subedi, Pushpa Subedi, Manikandan Panchatcharam, Hong Sun

**Affiliations:** Department of Cellular Biology and Anatomy, LSU Health Shreveport, Shreveport, LA 71103, USA; jiyu.li@lsuhs.edu (J.L.); chun.li@lsuhs.edu (C.L.); utsab.subedi@lsuhs.edu (U.S.); pushpa.subedi@lsuhs.edu (P.S.); manikandan.panchatcharam@lsuhs.edu (M.P.)

## Error in Figure 1

In the original publication [1], there were three mistakes in Figures 1, 4 and 5, as published. In Figure 1B of our submitted manuscript, two purple bars were used to indicate the “With AT-56” condition. However, the purple color was omitted in the final published version due to technical error. The corrected Figure 1 appears below.

**Figure 1:**
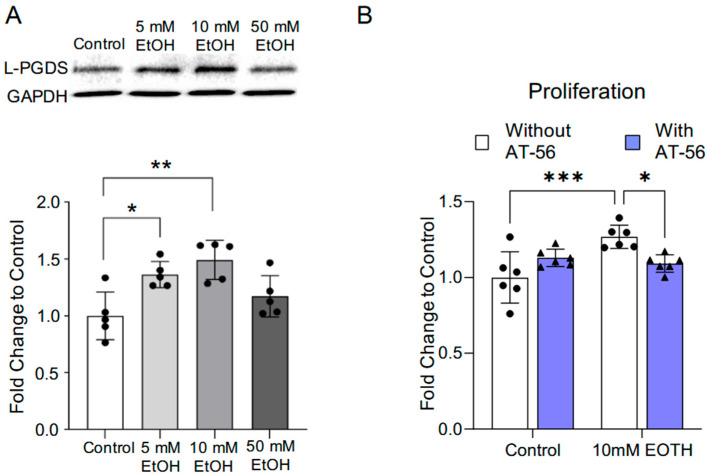
The influence of chronic alcohol exposure on L-PGDS protein expression and proliferation in MBMVECs. (**A**) Representative Western blots of L-PGDS (upper). Values are means ± SD (lower, *n* = 5). * *p* < 0.05, ** *p* < 0.005. Analyzed using one-way ANOVA with Dunnett’s post hoc. (**B**) Values are means ± SD (*n* = 6) for proliferation in the Proliferation Assay. * *p* < 0.05, *** *p* < 0.0005. Analyzed using two-way ANOVA followed by Tukey’s test.

## Error in Figure 4

In Figure 4, there was an extra copy of Western blots between Figure 4A and B. We inadvertently included an extra copy of the Western blot in the figure submitted for publication due to an oversight. The corrected Figure 4 appears below.

**Figure 4:**
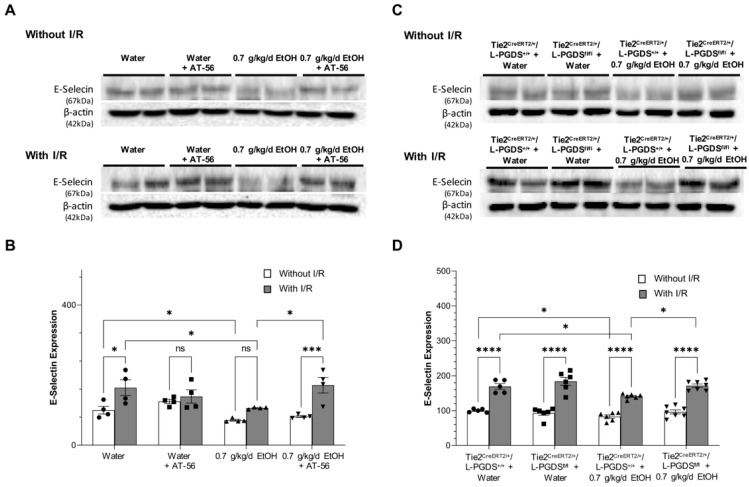
The effect of AT-56 and EC-specific L-PGDS knockout on E-selectin expression in the cerebral cortex under physiological conditions and following ischemic stroke. (**A**,**C**) Representative Western blots. (**B**,**D**) Values are means ± SD for 4–7 mice in each group. * *p* < 0.05, *** *p* < 0.0005, **** *p* < 0.0001. Analyzed using two-way ANOVA followed by Tukey’s test.

## Error in Figure 5

In Figure 5C, the white arrows changed to white squares. In the figure we submitted for publication, white arrows were used to indicate infiltrated neutrophils. However, in the final published version, these arrows were mistakenly replaced with squares due to a technical error. The corrected Figure 5 appears below.

**Figure 5:**
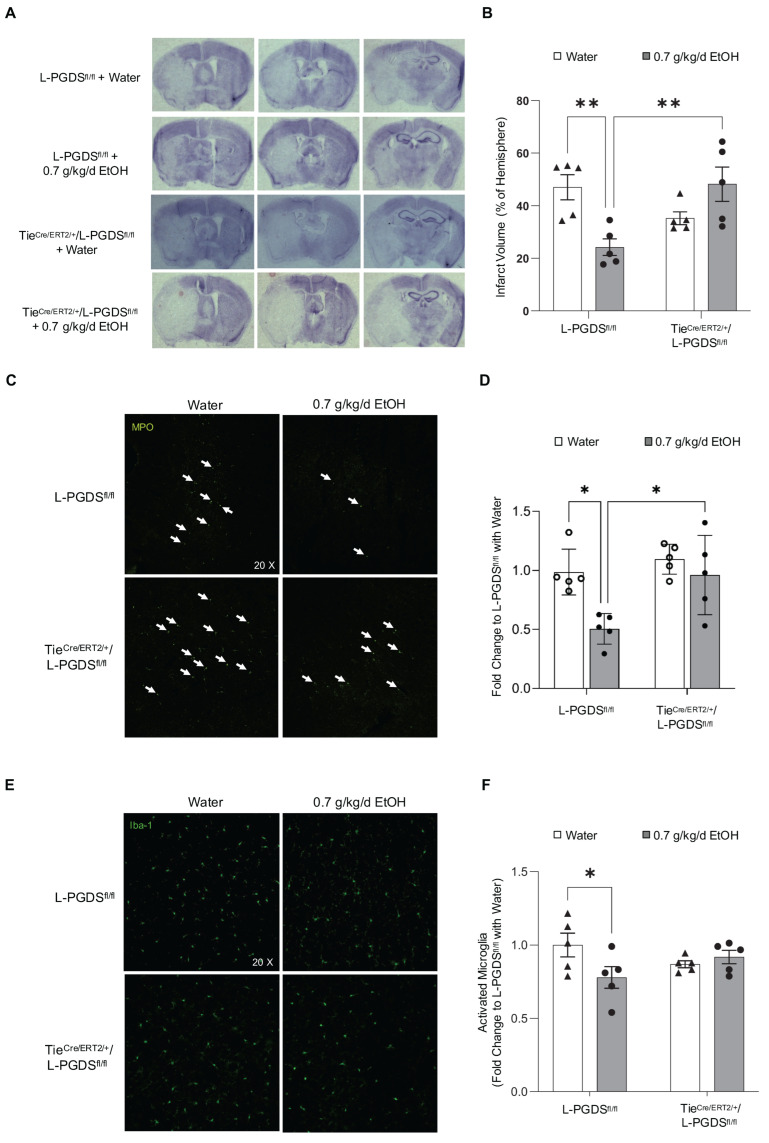
The effect of EC-specific L-PGDS knockout on infarct size and neutrophil infiltration and microglial activation in the peri-infarct cortex at 24 h of reperfusion following a 90 min MCAO. (**A**) Representative brain sections stained with cresyl violet. (**B**) Values are means ± SD for 5 mice in each group. (**C**) Representative immunohistochemistry staining of MPO. (**D**) Values are means ± SD for 5 mice in each group. (**E**) Representative immunohistochemistry staining of Iba1. (**F**) Values are means ± SD for 5 mice in each group. * *p* < 0.05, ** *p* < 0.005. Analyzed using two-way ANOVA followed by Tukey’s test.

The authors state that the scientific conclusions are unaffected. This correction was approved by the Academic Editor. The original publication has also been updated.
